# Acceptability, feasibility, and likelihood of stakeholders implementing the novel BPaL regimen to treat extensively drug-resistant tuberculosis patients

**DOI:** 10.1186/s12889-021-11427-y

**Published:** 2021-07-16

**Authors:** S. E. J. van de Berg, P. T. Pelzer, A. J. van der Land, E. Abdrakhmanova, A. Muhammad Ozi, M. Arias, S. Cook-Scalise, G. Dravniece, A. Gebhard, S. Juneja, R. Handayani, D. Kappel, M. Kimerling, I. Koppelaar, S. Malhotra, B. Myrzaliev, B. Nsa, J. Sugiharto, N. Engel, C. Mulder, S. van den Hof

**Affiliations:** 1 KNCV Tuberculosis foundation, The Hague, The Netherlands; 2 National TB Program Kyrgyzstan, Bishkek, Kyrgyzstan; 3 National Tuberculosis and Leprosy control Program Nigeria, Mabushi, Nigeria; 4 TB Alliance, New York, USA; 5 PATH, Kyiv, Ukraine; 6 National TB Program Indonesia, Jakarta, Indonesia; 7 KNCV country office Kyrgyzstan, Bishkek, Kyrgyzstan; 8 KNCV country office Nigeria, Abuja, Nigeria; 9 Yayasan KNCV Indonesia, Jakarta, Indonesia; 10 Maastricht University, Maastricht, The Netherlands; 11 Amsterdam Institute for Global Health and Development, Amsterdam University Medical Center, Amsterdam, The Netherlands; 12 National Institute for Public Health and the Environment, Bilthoven, The Netherlands

**Keywords:** BPaL, Pretomanid, XDR-TB, Novel TB regimen, Acceptability, Feasibility, Implementation

## Abstract

**Background:**

BPaL, a 6 month oral regimen composed of bedaquiline, pretomanid, and linezolid for treating extensively drug-resistant tuberculosis (XDR-TB) is a potential alternative for at least 20 months of individualized treatment regimens (ITR). The ITR has low tolerability, treatment adherence, and success rates, and hence to limit patient burden, loss to follow-up and the emergence of resistance it is essential to implement new DR-TB regimens. The objective of this study was to assess the acceptability, feasibility, and likelihood of implementing BPaL in Indonesia, Kyrgyzstan, and Nigeria.

**Methods:**

We conducted a concurrent mixed-methods study among a cross-section of health care workers, programmatic and laboratory stakeholders between May 2018 and May 2019. We conducted semi-structured interviews and focus group discussions to assess perceptions on acceptability and feasibility of implementing BPaL. We determined the proportions of a recoded 3-point Likert scale (acceptable; neutral; unacceptable), as well as the overall likelihood of implementing BPaL (likely; neutral; unlikely) that participants graded per regimen, pre-defined aspect and country. We analysed the qualitative results using a deductive framework analysis.

**Results:**

In total 188 stakeholders participated in this study: 63 from Kyrgyzstan, 51 from Indonesia, and 74 from Nigeria The majority were health care workers (110). Overall, 88% (146/166) of the stakeholders would likely implement BPaL once available. Overall acceptability for BPaL was high, especially patient friendliness was often rated as acceptable (93%, 124/133). In contrast, patient friendliness of the ITR was rated as acceptable by 45%. Stakeholders appreciated that BPaL would reduce workload and financial burden on the health care system. However, several stakeholders expressed concerns regarding BPaL safety (monitoring), long-term efficacy, and national regulatory requirements regarding introduction of the regimen. Stakeholders stressed the importance of addressing current health systems constraints as well, especially in treatment and safety monitoring systems.

**Conclusions:**

Acceptability and feasibility of the BPaL regimen is high among TB stakeholders in Indonesia, Kyrgyzstan, and Nigeria. The majority is willing to start using BPaL as the standard of care for eligible patients despite country-specific health system constraints.

**Supplementary Information:**

The online version contains supplementary material available at 10.1186/s12889-021-11427-y.

## Background

Despite increasing access to new diagnostics and anti-tuberculosis (TB) medicines, treatment success rates for extensively drug-resistant TB (XDR-TB) remain low and contribute to the ongoing transmission of DR-TB [1]. Globally, health systems have challenges to effectively implement DR-TB treatment regimens, resulting in sub-optimal overall treatment success rates and increasing resistance [2]. At the time of the study (2018–2019), XDR-TB was treated using a 20-months treatment consisting of at least six drugs (Individualized Treatment Regimen (ITR)) [3]. During treatment patients were recommended to be examined at least monthly to reduce the risk and impact of drug side effects. In many countries, XDR-TB patients were hospitalized for the full duration of treatment. To reduce the incidence of XDR-TB and to reach the 2030 End TB targets, shorter and more effective DR-TB treatment regimens are nessesary [4].

The Global Alliance for TB Drug Development (TB Alliance) has developed a 6 month oral regimen consisting of three drugs: bedaquiline (Bdq), the new drug pretomanid, and linezolid (Lzd). This BPaL regimen was successful in over 90% of patients with XDR-TB, multidrug resistant TB (MDR-TB) treatment failure, or intolerance to MDR-TB treatment in the Nix-TB trial [5]. Side effects of BPaL reported in this trial were: myelosuppression, peripheral neuropathy, optic neuritis (all three related to linezolid), QT prolongation (bedaquiline and possibly pretomanid) and hepatotoxicity (bedaquiline and pretomanid) [5]. The WHO endorsed the use of BPaL under operational research conditions for the treatment of XDR-TB, fluoroquinolone-resistant and treatment failure/treatment intolerant TB [6]. The United States Food and Drug Administration, and European Medicines Agency approved pretomanid as part of the BPaL regimen [7, 8].

Implementation of the BPaL regimen would entail essential changes in clinical and programmatic TB management [9]. The reduction of treatment duration from 20 months to 6 months would entail a potentially significant improvement of patient comfort and retention. Especially DR-TB treatment is associated with severe side effects and financial constraints for patients as a result of treatment costs and loss of work [10]. It may also reduce the financial burden on the health care system as less resources may be required for the treatment itself, health monitoring during treatment and human resource capacity. Implementation of the novel regimen may also entail adjusted patient follow-up and health monitoring during treatment and consequent changes in infrastructure and human resource capacity. It may also require a reorganization of established procurement and supply chain mechanisms.

To ensure rapid adoption after the regimen is endorsed by regulatory authorities, evidence is needed on health care system readiness, barriers and requirements among those implementing the intervention [11–13]. The objective of this study was to assess the acceptability and feasibility of implementing BPaL in eligible treatment groups among stakeholders in Programmatic Management of DR-TB (PMDT).

## Methods

### Study design

We used a concurrent mixed methods design. The qualitative component, consisting of semi-structured interviews and focus group discussions (FGD) among stakeholders, explored the acceptability and feasibility of seven aspects of PMDT, comparing ITR and BPaL. The seven aspects of PMDT were: patient baseline assessment and monitoring of treatment efficacy, treatment safety monitoring, patient friendliness, patient support, programmatic aspects, human resources, and procurement and supply chain management (PSCM).

For the quantitative component of the study, we used a Likert scale to quantify how acceptable stakeholders perceived each PMDT aspect for ITR and BPaL, as well as the overall likelihood of implementing BPaL. We triangulated the two data types to explore if quantitative findings could be explained by the qualitative results.

### Study population and setting

We conducted the study from May 2018 – May 2019 among health care workers (general, private, and public hospital clinicians and nurses; specialized MDR-TB treatment clinicians, nurses, and case managers), programmatic stakeholders (patient advocates, policy makers, budget owners, national guideline developers, PSCM staff, international experts, donors, and technical partners) and laboratory stakeholders (national reference laboratory managers, and laboratory managers from public and private laboratories) in Indonesia, Kyrgyzstan, and Nigeria. Target participant numbers per subgroup are shown in Supplementary file 1. Rather than drawing a statistically representative sample, we conveniently sampled eligible participants aiming to obtain a wide range of views. We thereby drew upon established local relationships while at the same time aiming to ensure diversity in geographies and capacity of health facilities. We collected qualitative and quantitative data concurrently in the same population sample.

We chose Indonesia, Kyrgyzstan, and Nigeria to depict various geographical settings (South-East Asia, Central Asia, Africa), with differences in TB burden (i.e., mixture of Drug-susceptible- (DS) and DR-TB) and health systems infrastructure. The estimated TB incidence per 100,000 population in 2018 was 316 in Indonesia, 116 in Kyrgyzstan and 219 in Nigeria [14]. The estimated MDR/RR-TB incidence per 100,000 population ranged from 8.8 in Indonesia, to 11 in Nigeria and 47 in Kyrgyzstan. Indonesia’s health system infrastructure greatly relies on the private sector which is involved in TB diagnosis and treatment at primary and secondary care level [15, 16]. In Kyrgyzstan, by contrast, TB-care is organized vertically and only provided by state-owned facilities [17, 18]. In Nigeria, TB-services are provided through a mix of public and private services, though interconnections were reported to be weak [19].

**Fig. 1:**
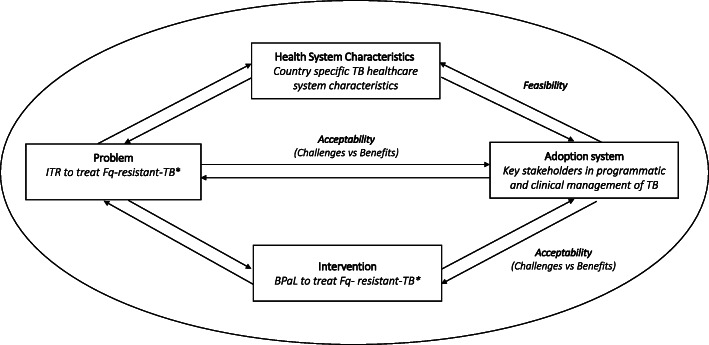
Conceptual framework of factors influencing implementation of BPaL in an existing health care system [11]. *Specified along assessment aspects: Baseline assessment and monitoring of treatment efficacy; Treatment safety monitoring; Patient friendliness; Patient support; Programmatic aspects; Human resources; PSCM. BPaL: bedaquilline & pretomanid & linezolid, Fq: fluoroquinolone, ITR: Individualized Treatment Regimen, PSCM: Procurement and supply chain management, TB: tuberculosis

The conceptual framework from Atun et al. [11] guided the design and analysis of this study. The framework maps factors that influence the process of integrating new interventions into health systems (Fig. 1). We explored the “problem” by assessing the acceptability of DR-TB management with the currently available DR TB treatment by asking benefits and challenges regarding DR-TB management with the current ITR (Fig. 1). We explored the acceptability of the “intervention” by assessing the anticipated benefits and challenges regarding DR TB management with the BPaL regimen by the “adoption system” represented by the stakeholder groups. In addition, we explored the feasibility of the intervention by assessing stakeholders’ expectations regarding the practical requirements for implementing the BPaL regimen within the context of their specific “health system characteristics”. The fifth element of Atun’s framework, the broader non-TB health systems context, was beyond the scope of this study.

We developed the interview guide and questionnaire (Supplementary file 2) based on the study’s conceptual framework and the defined PMDT aspects. We piloted all data collection tools among nine stakeholders in Nigeria who participated in an FGD and completed the questionnaire. Subsequently, we adjusted the interview guide and questionnaire according to participants’ feedback. Piloting was done in Nigeria because this allowed the English-speaking researchers to conduct the pilot without translation Project teams translated data collection tools in Indonesia and Kyrgyzstan into Bahasa and Russian, respectively. Locally trained consultants in the respective countries conducted the interviews. The local consultant in Kyrgyzstan provided on-site translation into local language as some of the interviewees were only conversant in the local Kyrgyz language.

### Qualitative data

We conducted 9 semi-structured interviews in Kyrgyzstan, 9 in Indonesia, and 16 in Nigeria, among stakeholders who were in senior positions at the National TB Program and national regulatory authorities. We stopped interviews after we achieved data saturation. In Nigeria more interviews were needed to reach this. We conducted 10 FGDs in Kyrgyzstan, 10 in Indonesia, and 10 in Nigeria, with 3–8 participants each, among stakeholders from heterogeneous levels of care and separated stakeholders in a supervisory position from their supervisees to avoid biases induced by hierarchical structures.

As per the interview guide, we asked participants to describe benefits and challenges of the ITR as compared to BPaL for the PMDT aspects as well as perceived requirements for the implementation of BPaL. We adjusted the PMDT aspects addressed in the interviews and FGD to the stakeholders’ fields of expertise (Supplementary file 1). To that end we did not explicitly question case managers and patient advocacy representatives on PSCM; PSCM staff on treatment monitoring, patient friendliness and programmatic aspects; international experts, technical partners and donors on patient support, human resources and PSCM; and laboratory stakeholders on patient friendliness, programmatic aspects and PSCM.

Interviews and FGDs were audio-recorded, transcribed verbatim, and translated into English by a local translator and PMDT consultant in Indonesia and Kyrgyzstan. All transcripts were quality-checked by the local PMDT consultants.

We used a deductive thematic framework analysis [20]. We coded the semi-structured interviews and FGDs by using a predefined coding scheme based on the study’s PMDT aspects and conceptual framework. To ensure intercoder validity, all researchers coded one interview in parallel and resolved discrepancies through discussion and adjustments. Results were summarized per PMDT aspect, taking into account the role of the respondent making the statement and the emphasis placed on the statement. We used NVivo (QSR International Pty Ltd. Version 12, 2018) to assist in the analysis.

### Quantitative data

Participants scored the acceptability of BPaL and ITR of each aspect covered during the semi-structured interviews (Supplementary file 1) on a 5 -point Likert scale (very acceptable, acceptable, neutral, unacceptable, very unacceptable), (Supplementary file 3). Additionally, they scored on a 1 to 5 scale (very unlikely, unlikely, neutral, likely, very likely) the likelihood of implementing BPaL as the Standard of Care (SoC) in two scenarios: 1) Treatment of patients with XDR-TB and MDR-TB treatment failure/intolerance, and 2) Treatment of MDR-TB patients with fluoroquinolone (FQ) resistance regardless of additional resistance to second line injectables (SLI) (Supplementary file 3). We collected the quantitative data immediately after the semi-structured interview/FGDs to build upon participants’ qualitative assessments. We entered all quantitative data into SPSS (IBM Corp. Released 2017, IBM SPSS Statistics for Windows, Version 25.0, Armonk, NY: IBP Corp) and recoded the 5-point Likert scale into 3 categories (very acceptable and very unacceptable merged with acceptable and unacceptable, respectively).

We determined the proportions of participants that chose acceptable, neutral and not acceptable per regimen (ITR vs BPaL) and PMDT aspect. We also determined the proportion of stakeholder that chose likely, neutral and not likely per scenario of implementing BPaL as SoC. We stratified results by country.

## Results

A total of 194 stakeholders participated in this study. Four Kyrgyz participants were excluded from all analyses due to inaudible recordings and two Kyrgyz participants were excluded due to declined consent for recording. Three stakeholders in Indonesia and three in Nigeria did not fill out the questionnaire for the quantitative part. Thus in total, 188 stakeholders were included in the qualitative data analysis and 182 in the quantitative analyses (97 and 94% respectively) (Table 1).

**Table 1:** Study participants categories in Indonesia, Kyrgyzstan, Nigeria and all countries combined

	Qualitative part				Quantitative part			
	Total	Indonesia^**a**^	Kyrgyzstan	Nigeria	Total	Indonesia^**a**^	Kyrgyzstan	Nigeria
**Total**	188	51	63	74	182	48	63	71
**Health care workers**	110	28	44	38	110	28	44	38
**Laboratory stakeholders**	78	23	12	9	72	20	12	9
**Programmatic Stakeholders**			7	27			7	24

^a^The categories laboratory and programmatic stakeholders were merged to ensure anonymity

### Quantitative findings

#### Acceptability ratings

The aggregated acceptability from all three countries as well as the country specific values for all categories assessed ranged from 79 to 93% for BPaL and 45 to 77% for the ITR (Table 2).

**Table 2:** Acceptability ratings for aspects of the BPaL (Nix) regimen and the individualized treatment regimen^a^

		Overall (***N*** = 182)		Indonesia		Kyrgyzstan		Nigeria	
		ITR	BPaL	ITR	BPaL	ITR	BPaL	ITR	BPaL
**Baseline assessment and monitoring of treatment efficacy**	**Total**	144	144	46	45	51	50	47	49
	**Acceptable**	106 (74%)	127 (88%)	35 (76%)	38 (84%)	43 (84%)	47 (94%)	28 (60%)	42 (86%)
	**Neutral**	26 (18%)	14 (10%)	7 (15%)	7 (16%)	6 (12%)	2 (4%)	13 (28%)	5 (10%)
	**Unacceptable**	12 (8%)	3 (2%)	4 (9%)	0 (0%)	2 (4%)	1 (2%)	6 (13%)	2 (4%)
**Treatment safety monitoring**	**Total**	141	140	44	43	52	52	45	45
	**Acceptable**	108 (77%)	115 (84%)	39 (89%)	31 (72%)	42 (81%)	45 (87%)	27 (60%)	39 (87%)
	**Neutral**	19 (14%)	21 (15%)	2 (5%)	9 (21%)	5 (10%)	7 (14%)	12 (27%)	5 (11%)
	**Unacceptable**	14 (10%)	4 (3%)	3 (7%)	3 (7%)	5 (10%)	0 (0%)	6 (13%)	1 (2%)
**Patient friendliness**	**Total**	136	133	43	43	39	39	54	51
	**Acceptable**	61 (45%)	124 (93%)	20 (47%)	37 (86%)	23 (59%)	37 (95%)	18 (33%)	50 (98%)
	**Neutral**	33 (24%)	6 (5%)	11 (26%)	5 (12%)	9 (23%)	1 (3%)	13 (24%)	0 (0%)
	**Unacceptable**	42 (31%)	3 (2%)	12 (28%)	1 (2%)	7 (18%)	1 (3%)	23 (43%)	1 (2%)
**Programmatic aspects**	**Total**	141	132	44	42	41	41	56	53
	**Acceptable**	85 (60%)	110 (83%)	30 (68%)	29 (69%)	28 (68%)	33 (89%)	27 (48%)	48 (91%)
	**Neutral**	32 (23%)	19 (14%)	8 (18%)	11 (26%)	10 (24%)	3 (8%)	14 (25%)	5 (9%)
	**Unacceptable**	24 (17%)	3 (2%)	6 (14%)	2 (5%)	3 (7%)	1 (3%)	15 (27%)	0 (0%)
**Patient support**	**Total**	146	144	41	41	48	48	57	54
	**Acceptable**	100 (69%)	122 (85%)	29 (71%)	33 (81%)	34 (71%)	45 (92%)	37 (65%)	44 (82%)
	**Neutral**	27 (19%)	19 (13%)	9 (22%)	8 (20%)	6 (13%)	3 (6%)	12 (21%)	8 (15%)
	**Unacceptable**	19 (13%)	3 (2%)	3 (7%)	0 (0%)	8 (17%)	1 (2%)	8 (14%)	2 (4%)
**Human resources**	**Total**	153	153	42	42	49	51	62	60
	**Acceptable**	90 (59%)	121 (79%)	29 (69%)	27 (64%)	29 (59%)	46 (90%)	32 (52%)	48 (80%)
	**Neutral**	43 (28%)	26 (17%)	9 (21%)	13 (31%)	14 (29%)	4 (8%)	20 (32%)	9 (15%)
	**Unacceptable**	20 (13%)	6 (4%)	4 (10%)	2 (5%)	6 (12%)	1 (2%)	10 (16%)	3 (5%)
**PSCM**	**Total**	79	74	25	25	28	26	26	24
	**Acceptable**	53 (67%)	59 (80%)	18 (72%)	14 (56%)	20 (71%)	23 (92%)	15 (58%)	22 (92%)
	**Neutral**	14 (18%)	13 (18%)	4 (16%)	10 (40%)	5 (18%)	2 (8%)	5 (19%)	1 (4%)
	**Unacceptable**	12 (15%)	2 (3%)	3 (12%)	1 (4%)	3 (11%)	1 (0%)	6 (23%)	1 (4%)

^a^ Since certain topics of the acceptability matrix were only collected for certain stakeholder groups and optional or not required for others (Table 1), only missing values for “mandatory” topics for the respective stakeholder were considered “true missing” values. Answers were excluded if the question was not a topic for the respective stakeholder

*BPaL* bedaquiline & pretomanid & linezolid, *ITR* Individualized Treatment Regimen, *PSCM* procurement and supply chain management

Compared to the ITR, Kyrgyz, and Nigerian stakeholders rated BPaL more often acceptable on all aspects. Indonesian stakeholders rated BPaL acceptable more often or comparable to ITR for all categories, except for the category of PSCM and treatment safety monitoring. In all three countries, the acceptability of patient friendliness differed most between the ITR and BPaL, ranging from 47, 59 and 33% acceptable for ITR compared to 86, 94, and 98% for the BPaL regimen in Indonesia, Kyrgyzstan, and Nigeria, respectively. Unacceptability scores for BPaL were lower than ITR for all aspects in all countries.

The proportion of stakeholders rating the ITR unacceptable, ranged from 7 to 28% in Indonesia from 4 to 18% in Kyrgyzstan, and from 13 to 45% in Nigeria. The proportion of stakeholders finding BPaL unacceptable was lower than ITR in all countries.

#### Likelihood of implementation

The majority of stakeholders across the three countries indicated that they would be likely to implement BPaL based on its initial profile (Nix regimen dosing [4]) as the SoC for treatment of XDR-TB and MDR-TB treatment failure/intolerance (88%) and for treatment of FQ resistant TB without additional resistance to SLI (84%) (Table 3). The likelihood of implementation of BPaL as the SoC for treatment of FQ resistant TB without additional resistance to SLI was slightly lower in all three countries compared to the implementation for treatment of XDR-TB and MDR-TB treatment failure/intolerance.

**Table 3:** Likelihood of implementation of the BPaL (Nix) regimen as standard of care

		Overall	Indonesia	Kyrgyzstan	Nigeria
		n (%)	n (%)	n (%)	n (%)
Implementation of BPaL as the SoC for treatment of XDR-TB and MDR-TB treatment failure/intolerance based on initial profile (Nix)	**Total**	166	46	50	70
	**Likely**	146 (88%)	40 (87%)	43 (86%)	63 (90%)
	**Neutral**	18 (11%)	6 (13%)	7 (14%)	5 (7%)
	**Unlikely**	2 (1%)	0 (0%)	0 (0%)	2 (3%)
Implementation of BPaL as SoC for treatment of Fq resistant TB without additional resistance to 2nd line injectables based on initial profile (Nix)	**Total**	169	46	55	68
	**Likely**	142 (84%)	38 (83%)	44 (80%)	60 (88%)
	**Neutral**	20 (12%)	8 (17%)	10 (18%)	2 (3%)
	**Unlikely**	7 (4%)	0 (0%)	1 (2%)	6 (9%)

*BPaL* bedaquilline & pretomanid & linezolid, *Fq* fluoroquinolone, *MDR* multi-drug resistant, *SoC* standard of care, *TB* tuberculosis, *XDR* extensively drug-resistant

### Qualitative findings

#### Patient-friendliness

In terms of benefits, stakeholders perceived BPaL to be more patient-friendly compared to the ITR resulting from its shorter treatment duration, lower pill burden per day, fewer anticipated side effects, lower financial burden, decreased requirements for higher level of care, and reduced patient discomfort (including risk of hearing loss) due to the removal of injectables (Table 4).

*Nigerian health care worker: “ … (compared to the) individualized treatment regimen (there are many) benefits are … no matter how somebody tries, taking a drug for 20 months is not easy … in terms of adherence, … out of pocket cost, … side effects … (with the 6 months regimen) they (the patients) are going to be treated quickly and … resume their normal economic activities … I think there are more benefits for them.” [FGD]*

Another benefit was that stakeholders believed that the BPaL regimen would increase patients’ quality of life during treatment and would improve treatment adherence, which would contribute to higher treatment success (Table 4).

*Indonesian caregiver: “Side effects (of BPaL are) … much less … compared to the individualized (regimen) … so for the patient’s level of … adherence … perhaps it’d be better because what happens so far, the patient cannot bear the side effects and becomes lost to follow up... he stops taking the medication.” [interview]*

Besides the reduction of side effects being a benefit, a challenge participants opted was about the side effects of (high-dose) Lzd, including myelosuppression (bone marrow suppression (which leads to reduced production of blood cells) and peripheral neuropathy (damage to the peripheral nerves which may cause weakness, numbness and pain) (Table 4). Stakeholders worried that the side effects of (high-dose) Lzd may be too harmful to the patients’ health:

*Nigerian health care worker: “ … this Linezolid is another challenge. I thought they were going to remove it completely, because the effect is too much. You know patients are really complaining, … eye pain, leg pain … , they are crying, they can’t sleep and before you know, by the time you do the PCV (packed cell volume), it is very low the patient is dying slowly...” [FGD]*

**Table 4:** Perceived benefits and challenges regarding the treatment of (X)DR-TB with the novel BPaL regimen and practical requirements for implementation

		Indonesia	Kyrgyzstan	Nigeria
**Acceptability**	**Anticipated benefits of the BPaL regimen**	• Shorter duration of treatment • Lower pill burden • Absence of injectables • Reduction AEs • Shorter duration of AEs • Expected reduction of treatment costs • Expected reduction of healthcare facility visits • Expected increase in quality of life for patients • Expected increase in treatment adherence		
		Expected increase likelihood to undergo treatment Expected reduction of financial burden on patient and health system Increased treatment success also in PLHIV Currently low resistance to the drugs in the regimen	Expected reduction of workload for HW Existing experience with Bdq and Lzd Expected reduction of TB transmission	• Expected reduction of hospitalization • Absence of risk of hearing loss • Expected reduction of workload for HW in hospitals • Possibility of decentralization of treatment • Improvement of treatment outcomes • Expected easier PSCM • Expected reduction of financial burden on the patient
	**Challenges related to overall system barriers for effective M/XDR TB management**	• Concerns regarding lack of capacity for monitoring and management of AE’s, especially in ambulatory care settings • High rate of LTFU		
		Relatively high price of locally procured Lzd Lack of DST capacity for Lzd and Bdq In some areas insufficient access to Xpert and SL LPA		• Lack of health insurance coverage • Lack of coverage of monitoring tests • Insufficient access to Xpert, SL LPA, • Lack of DST capacity for Lzd and Bdq • Insufficient patient and transportation support • Lack of ancillary drugs • Lack of attention to DOT • Lack of community infection control measures
	**BPaL regimen specific concerns**	• Concerns about AEs related to high dose Lzd • Concerns about generalizability of Nix study results to local population, pregnant women, children • Lack of DST capacity for Pa		
		Concerns about interaction between Pa and ARV drugs	• Uncertainties about BPaL treatment among patients with comorbidities • Worries about possible high price of Pa • Worries about lack of salvage regimen	• Lack of experience with Pa among clinicians • Worries about possible high cost •Worries about resistance development for BPaL especially if Lzd needs to be stopped
**Feasibility**	**Practical requirements for BPaL implementation**	• International recommendations for use, especially from WHO • Final study publications, including relapse rate • Additional evidence on pregnant women, children and in local populations • Capacity building / training for the monitoring and management of AEs • Ancillary drugs for management of AEs • Continuation of counseling, patient support and enablers		
		Development of capacity for DST for Bdq, Lzd, Pa Overall strengthening of programmatic management of DR-TB Strengthening of the laboratory system: increasing access to Xpert testing, SL PLA Community infection control in case of decentralization of treatment Ruling from the Advisory Committee to the MoH Innovative ways for DOT at the home of the patients (video etc.)	• Development of DST capacity for Pa • Hospitalization for some patients, good ambulatory management for others • Salvage regimen for failures of BPaL • Political involvement • Low price of Pa, especially in relation to transitioning of SLD’s to domestic budgets • Innovative ways for DOT at the home of the patients (video etc.)	• Development of capacity for DST for Bdq, Lzd, Pa • Overall strengthening of programmatic management of DR-TB • Overall strengthening of the laboratory system: increasing access to Xpert testing, SL PLA • Sufficient patient support / transportation • Well planned transition to more community-based treatment • Sufficient funding • Low price of Pa, for domestic and international procurement

*AE* adverse event, *ARV* anti-retroviral, *Bdq* Bedaquilline, *DOT* directly observed treatment, *DR* drug-resistant, *DST* drug susceptibility testing, *HW* health workers, *LTFU* loss to follow-up, *Lzd* Linezolid, *MoH* Ministry of Health, *Pa* pretomanid, *PLHIV* people living with HIV, *PSCM* procurement and supply chain management, *SLD* second-line drugs, *SL LPA* second-line line probe assay, *TB* tuberculosis

#### Patient support

Participants thought the BPaL regimen would allow cutting costs for patient support due to its shorter duration, while they thought the type/form of patient support required was the same (Table 4), including traditional forms of supervision as illustrated in the following quote:

*Indonesian health care worker: ( … ) we should pay attention to ( … ) supervise them (the patients) taking ( … ) the drugs … We have to provide counselling for the patients to remind them the importance of taking the drugs. It is the same.” [FGD]*

#### Programmatic aspects

Programmatic benefits of the ITR compared to BPaL included applicability of BPaL for children, and the inclusion of nutrition and monitoring in the ITR funding coverage (Table 5). The shorter treatment duration was considered to reduce financial burden on the health system and increase the possibility for the decentralization of treatment provision (Table 4). Others considered decentralization a challenge, due to the limited clinical management capacity at primary care, with regard to prevention of transmission and limited outpatient possibilities to ensure treatment adherence.

*Indonesian Programmatic Stakeholder: “The initial phase and this would be a challenge if it [BPaL] is (implemented) in Puskesmas [Indonesian primary health centres], in an outpatient setting … because we must still separate the patients that have been (tested) negative from the patients who are still positive..” [Interview]*

Despite the anticipated cost reduction for DR-TB treatment with BPaL, stakeholders from all countries considered current high costs of Bdq and Lzd a challenge (Table 4). Additionally, they were concerned about a possible manufacturer monopoly for pretomanid leading to high prices:

*Kyrgyz programmatic stakeholder:* “*If the manufacturer is a monopolist … the price will not be very low and it will not be affordable for everyone.” [Interview]*

Regarding practical requirements, some stakeholders emphasized the need for affordable drug prices, especially considering transition from the Global Fund to domestic funds for the procurement of second line drugs (Table 4). Depending on the type of funding (domestic vs. donor), some stakeholders stressed the need for local registration. Furthermore, political involvement would be crucial for the implementation of any novel regimen. Another practical requirement was that Kyrgyz and one Indonesian stakeholder favoured hospitalization for the full course of treatment, even with BPaL, to ensure close monitoring (Kyrgyz health care worker) and to support operational research conditions (Indonesian programmatic stakeholder).

**Table 5:** Perceived benefits and challenges regarding the current individualized (X) DR TB treatment

		Indonesia	Kyrgyzstan	Nigeria
**Acceptability**	**Perceived benefits regarding the current ITR**	• Proven efficacy according to WHO • Monitoring and management of AEs covered by health insurance • Enablers and nutrition provided with the regimen	• Reduced side effects of current ITR compared to the one before • Good completion rates • No resistance yet	• Use in children possible • Funding for drugs through the Global Fund • Monitoring also funded
	**Perceived challenges regarding the current ITR**	• Long duration of treatment • High pill burden • High health worker and health facility workload • Side effects common • Injectables		
		• Difficulty in quantification and forecasting due to individual dosing	• Limited adherence • Difficulties with treatment monitoring • Difficulties in allocating treatment in children	• Injectables (resulting in AEs and high HR needs) • Hospitalization • High workload on home visits if home-based care setting • Diagnostic treatment gap due to lack of funding for travel for baseline investigations • Limited adherence • Lack of tests for all examinations for AEs • High cost for monitoring • Difficulty in quantification and forecasting due to individual dosing

*AE* adverse event, *ITR* individualized treatment regimen, *HR* human resources

#### Baseline assessment and treatment efficacy monitoring

In terms of benefits, stakeholders perceived baseline assessment and treatment efficacy monitoring for BPaL to be less burdensome and more cost-saving as BPaL requires fewer follow-up visits and bacteriological tests (Table 4). However, Nigerian programmatic stakeholders brought forward the challenge about the lack of capacity to perform cultures and chest X-rays if treatment were to be decentralized. In terms of practical requirements, Indonesian stakeholders expressed the need for more guidance regarding bacteriological follow-up, i.e. what bacteriological proof (i.e. culture sample) would be required during the 6 months and as follow-up to declare a patient cured.

#### Human resources

Anticipated reduced workload, which health care workers currently perceive to be high in DR-TB care, was found to be a strong benefit (Tables 4, 5). Another benefit indicated was the removal of injectables. This could, according to the stakeholders, considerably reduce the need for human resources, as it would reduce the amount of required home visits.

In terms of challenges, some stakeholders were concerned about potentially increased workload at decentralized levels, because of possible ambulatory BPaL treatment. Most stressed as a practical requirement the need for training on BPaL implementation, specifically, in identification of eligible patients, and monitoring of adverse events.

#### Procurement and supply chain management

Stakeholders considered simpler PSCM for BPaL as a benefit. In Indonesia and Nigeria, stakeholders experienced forecasting for the ITR as a challenge, which led to stock outs (Indonesia). Some Kyrgyz and Nigerian stakeholders had experienced supply challenges with the introduction of the shorter regimen (Nigeria, due to distribution difficulties), Bdq and Delamanid (due to lack of registration). They therefore had concerns about sufficient supply of new drugs.

#### Monitoring of treatment safety

For monitoring of treatment safety, stakeholders considered it a benefit that BPaL has shorter duration and less audiometry requirements. However, many also perceived challenges due to uncertainties about safety, possible need for intensified monitoring due to the high-dose of Lzd in the regimen and lack of experience with pretomanid:

*Kyrgyz health care worker: “So, its (safety) is unclear yet. For example, with linezolid we notice that side effects are irreversible and with pretomanid we don’t know are they reversible or irreversible... it is not studied yet … ” [Interview]*

Indonesian and Nigerian stakeholders expressed challenges about current health systems gaps, especially at lower health care levels where BPaL may be implemented. These challenges included limited availability of ancillary drugs (Indonesia) and currently weak monitoring systems for regular haematology and electrocardiogram (ECG) (Nigeria). Stakeholders opted as practical requirement that safety monitoring and side effect management should be guaranteed and provision of ancillary drugs should be free of charge:

*Nigerian programmatic stakeholder: “We will have to strengthen our system to ensure that patients’ baseline and follow up investigations are done … when they are due so that we will be able to pick up any side effect at an early stage. If that is done, it will make it (BPaL) more friendly, but when that is not done patients will run away from it.” [Interview]*

#### Other anticipated benefits and challenges

Although some participants perceived the current expected low prevalence of drug resistance to drugs in the BPaL regimen as beneficial, participants from all countries had considered the emergence of resistance to BPaL or its components as a challenge. Nigerian health care workers were also concerned about baseline Lzd resistance as the drug is widely used for other diseases. Other challenges mentioned were about dose related Lzd intolerance and efficacy of the regimen when continuing treatment with only two drugs contributing to resistance to the remaining drugs or weakened regimen efficacy:

*Nigerian health care worker: “In case we adopt the BPaL and somebody has a serious adverse event (so) that you need to discontinue the linezolid, then what will happen to the potency of the regimen? … If you need to discontinue one of the drugs, what happens to the efficacy of the regimen?” [FGD]*

Another major challenge expressed by Kyrgyz and Nigerian stakeholders was on safety concerns and the applicability of BPaL among children, pregnant women, and patients with comorbidities.

In Indonesia and Nigeria, the lack of diagnostics and Drug Susceptibility Testing (DST) capacity for regimen allocation was expressed to be an important health systems challenge. This included the lack of access to GeneXpert MTB/RIF and/or second-line line probe assay testing in some areas, and the lack of DST capacity for Bdq and Lzd with liquid culture or genetic sequencing on a large scale.

#### Other anticipated practical requirements

Stakeholders expressed the need for clinical studies in certain groups and in a national context. They expressed the need for adjusted treatment guidelines among specific groups:

*Kyrgyz programmatic stakeholder:* “*Every patient should have an individual approach. Seriously ill patients with side effects, with additional comorbidities they need a completely different approach.” [Interview]*

Moreover, stakeholders stressed the importance of introducing BPaL under operational research conditions. The need was expressed for clear local guidelines, capacity building, and training on the use of BPaL. Stakeholders flagged the need for training on monitoring and management of DR TB treatment, related adverse events, and guidance on dealing with patients who fail on a regimen. Clear guidelines on Treatment efficacy- and safety monitoring, management of adverse events, as well as patient support would be needed to support decentralized implementation.

Other crucial practical requirements expressed for BPaL implementation were the needs for diagnostic capacity for DR-TB, regimen design including the drug composition of only three drugs (Table 4). PSCM planning (including funding) should not only focus on BPaL, but also include ancillary drugs for management of AEs and access to baseline and monitoring tests. Related to the challenge stakeholders perceived regarding the lack of DST testing, stakeholders considered diagnostic and DST capacity a prerequisite for BPaL implementation. Kyrgyz stakeholders suggest introducing DST to pretomanid before enrolment on BPaL treatment regimen.

## Discussion

We assessed the acceptability and feasibility of implementing BPaL among key stakeholders in TB care and management from Indonesia, Kyrgyzstan, and Nigeria. Study participants found BPaL more acceptable than the ITR because of anticipated benefits such as improved patient-friendliness and reduced health system burden as a result of increased patient-friendliness, likelihood of improved adherence and anticipated reduced cost of BPaL. Additionally, stakeholders considered the implementation of BPaL feasible within the local health care infrastructure if efforts are undertaken to address practical requirements such as gaps in current treatment and safety monitoring systems that have become more apparent with introduction of recent new drugs and regimens as well as efficient diagnostic and DST capacity.

Triangulating the qualitative and quantitative results indicated higher overall patient-friendliness score for BPaL (93%) in comparison to the ITR (45%) could be linked to stakeholders’ anticipated patient challenges with the current ITR which will be improved with BPaL, i.e. shorter duration, absence of injectables, lower pill burden, and lower financial burden to the patient. The shortened treatment duration and reduced costs may explain the higher overall acceptability of BPaL patient support in comparison to the ITR (85% vs. 69%). The higher overall acceptability of programmatic aspects of BPaL (83%) as compared to the ITR (60%) might be linked to the anticipated benefits of improved treatment outcomes as well as reduced financial burden to the health system due to the shorter treatment duration and increased possibility for the decentralization of treatment provision. Higher acceptability scores for the baseline assessment and treatment efficacy monitoring may be driven by stakeholders perceiving BPaL as beneficial in terms of being less burdensome and cost-saving as compared to the ITR. The higher acceptability for human resources for BPaL (79% vs. 59%) could be linked to the benefit of reduced burden on health care workers. The higher acceptability of PSCM (BPaL 80% vs. ITR 67%) may be explained by stakeholders’ positive expectation that PSCM will become simpler, with only three drugs compared to the 15 in ITR. Acceptability ratings in Indonesia may be lower due to concerns about complexities relating to local regulatory requirements and approvals, along with requirements to procure linezolid locally at a very high price. Similar acceptability scores on the treatment safety monitoring on BPaL and ITR may be explained by the expected benefits such as shorter duration (resulting in less treatment monitoring visits and actions) being offset by challenges relating to BPaL safety, and current health systems gaps.

Our findings aligned with those of Thomas et al. [10], and Vega et al. [21], with stakeholders confirming that current DR-TB treatment negatively impact psychological well-being and may pose a heavy financial burden. Our study also supports findings from other studies that current poor treatment outcomes of DR-TB patients could be attributed to long treatment duration, drug toxicity, and high pill burdens of ITR [22–24]. Furthermore, insufficient health and social protection mechanisms create financial hardship due to patients’ inability to work during treatment, which is especially detrimental during lengthy DR-TB treatment [10]. Furthermore, it was considered an advantage that the BPaL regimen could accelerate decentralized DR-TB care under certain circumstances, which might be beneficial for reducing health system costs [25, 26].

Many stakeholders were especially concerned about the side effects of Lzd, including myelosuppression and neuropathy. The ZeNix trial is assessing the efficacy, safety and tolerability of various doses and durations of Lzd in the BPaL regimen [27]. Consequently, those study results, along with future evidence on the use of BPaL in pregnant women, children, patients with comorbidities and local populations, likely will have a major impact on the acceptability of BPaL.

The stakeholders showed concern regarding the potential emergence of resistance to novel drugs such as Bdq, which highlights the need for development of rapid DST for new medicines [28]. Current diagnostic and DST gaps, especially at lower levels of care, were also brought forward as a concern. This in line with reporting by WHO that diagnosis of XDR-TB remains a challenge in many settings [1]. The development and maintenance of capacity to perform DST for Bdq, Pa, and Lzd will be crucial to prevent regimen failure and unnecessary resistance generation in the future. Furthermore, BPaL should be embedded in the overarching national TB diagnosis and treatment algorithms DR-TB care and treatment monitoring capacity is in many settings only available in designated centralized treatment facilities [29–31], this challenge was confirmed by the stakeholders in this study. This underlines the importance for countries to pay attention to all aspects of the TB care cascade as mentioned in WHO (and other guiding documents).

This study has some limitations. We did not include patients in this study. We aimed to represent their experiences indirectly by interviewing health care workers. As per the design of the assessment, many questions regarding the BPaL regimen were of a hypothetical nature and participants did not have any practical experience with BPaL at the time they were interviewed. Therefore, there may have been misconceptions leading to biased perceptions around BPaL. To mitigate these biases we provided each participant with standardized information about BPaL based on the available evidence from the Nix-TB trial. We used convenience sampling for selection of our interviewees. We acknowledge that this method may lead to a non-random sample. We aimed to limit this bias by inviting participants based on a clear guidance on expertise and positions. Another limitation was that the data collection tools were only piloted in Nigeria, the translated tools were not piloted which could have caused misinterpretation due to lingual and cultural differences. Each country employed at least 1 but no more than 3 interviewers to conduct interviews. This may have caused variability, which we tried to minimize by training of interviewers and standardizing semi-structured questionnaires. We addressed this by having an interviewer present who speaks the local language and as we reached data saturation, we do not expect the variability to have limited the quality and richness of our qualitative data.

To our knowledge, this is the first study to assess the acceptability and feasibility of the implementation of the BPaL regimen compared to current treatment options, thus providing unique insights from a health care system perspective. The participants appreciated being engaged in identifying and discussing concerns on safety and capacity early on; the richness of the discussions and the findings highlighted the usefulness of early stakeholder engagement when developing implementation strategies for new drugs and regimens. Studies using similar methods could be relevant to conduct future implementation research for a variety of novel drug interventions. A broader perspective should be obtained by also assessing patient perspectives. This limitation notwithstanding, our study provides some baseline evidence that could be useful for the conduct of more robust studies on this, and related research topics.

## Conclusion

Our assessment provides rich information from different stakeholder groups on perceptions and expectations on the BPaL regimen. It shows that the adoption of BPaL in three high DR-TB burden countries will be accepted and feasible. BPaL could increase the treatment success rate and alleviate the individual and health care system burden of DR-TB. Active TB drug-safety monitoring and management is crucial to address concerns on linezolid safety. National strategic plans should incorporate both the identified and anticipated country-specific challenges and requirements to facilitate smooth roll out of the BPaL regimen.

## Supplementary Information

**Additional file 1.**

**Additional file 2.**

**Additional file 3.**

## Data Availability

The datasets generated and analyzed during the current study are not publicly available as it contains information that could compromise the research participants. Parts of the data are available from the corresponding author on reasonable request, but restrictions apply.

## Abbreviations

BPaL: Bedaquiline, Pretomanid and Linezolid
Bdq: Bedaquiline
DR: Drug-resistant
DS: Drug-susceptible
DST: Drug-susceptibility test
ECG: Electro cardiogram
FQ: Fluoroquinolones
FGD: Focus group discussion
ITR: Individualized treatment regimen
Lzd: Linezolid
MDR: Multi-drug resistant
MTB: *Mycobacterium tuberculosis*
PMDT: Programmatic management of DR-TB
PSCM: Procurement and supply chain management
RIF: Rifampicin
RR: Rifampicin Resistant
SLI: Second line injectables
SoC: Standard of care
TB: Tuberculosis
WHO: World Health Organization
XDR: Extensively Drug-resistant
