# Supplementary material for: Acceptability, feasibility, and likelihood of stakeholders implementing the novel BPaL regimen to treat extensively drug-resistant tuberculosis patients
Source: BMC Public Health. 2021 Jul 16;21:1404. doi: 10.1186/s12889-021-11427-y (PMC8284025; doi:10.1186/s12889-021-11427-y)
Supplement: Supplementary file 1 — Additional file 1. [file 12889_2021_11427_MOESM1_ESM.docx]

**Supplementary Material 1****:. Stakeholder categories and assigned data collection method and assessment topics**

| **Aggregated stakeholder group** | **Type of stakeholder** | | **Type of assessment** | **Topics (incl. optional)** |
| --- | --- | --- | --- | --- |
| Health care workers | Clinicians from general public HCF level 1-3 | | FGD | 1-7 |
|  | Clinicians from general private HCF level 1-3 | | FGD | 1-7 |
|  | Nurses from general public HCF level 1-3 | | FGD | 1-7 |
|  | Nurses from general private HCF level 1-3 | | FGD | 1-7 |
|  | Specialized MDR treatment center clinicians | | FGD | 1-7 |
|  | Case managers | | Interview | 1-6 |
| Programmatic stakeholders | Patient advocacy representatives | | Interview | 1-6 |
|  | Policy makers & budget owners | MoH representatives | Interview | 1-7 |
|  |  | District health officers | FGD | 1-7 |
|  |  | Provincial health officers | FGD | 1-7 |
|  | National guideline development | NTP manager | Interview | 1-7 |
|  |  | DR-TB experts | Interview | 1-7 |
|  | PCSM unit staff | | Interview | 4,5,7 |
|  | International experts | WHO experts | Interview | 1,2,3,6 |
|  |  | GLC experts | Interview | 1,2,3,6 |
|  | Technical partners | | Interview | 1,2,3,6 |
|  | Donor (GF representatives) | | Interview | 1,2,3,6 |
| Laboratory Stakeholders | NRL manager | | FGD | 1,2,4,5 |
|  | Laboratory managers from public health care level 1-3 laboratories | | FGD | 1,2,4,5 |
|  | Laboratory managers from private health care level 1-3 laboratories | | FGD | 1,2,4,5 |

^DR-TB: Drug-resistant Tuberculosis, FGD: Focus Group Discussion, GF: Global Fund (The Global Fund to Fight AIDS, Tuberculosis and Malaria), GLC: Green Light Committee, HCF: Health Care Facility, MDR-TB: Multi-drug resistant Tuberculosis, MoH: Ministry of Health, NRL: National Reference Laboratory, NTP: National Tuberculosis Program, PSCM: Procurement and Supply Chain Management,^

^Topics: 1: baseline assessment and treatment efficacy monitoring; 2: treatment safety monitoring; 3: patient friendliness; 4: patient support; 5: human resources; 6: programmatic aspects, 7:PSCM^
