# Supplementary material for: Acceptability, feasibility, and likelihood of stakeholders implementing the novel BPaL regimen to treat extensively drug-resistant tuberculosis patients
Source: BMC Public Health. 2021 Jul 16;21:1404. doi: 10.1186/s12889-021-11427-y (PMC8284025; doi:10.1186/s12889-021-11427-y)
Supplement: Supplementary file 2 — Additional file 2. [file 12889_2021_11427_MOESM2_ESM.docx]

Semi-structured Interview/ Focus Group Discussion Guide on

Perceptions on current TB regimens and possible implementation of novel regimens BPaMZ/BPaL

**Instructions for the interviewer:**

*Thank you for helping us to conduct the interviews and focus group discussions (FGDs). During the interviews we would like you to identify stakeholders’ perceptions on current TB regimens and on the potential implementation of the novel regimens BPaMZ for DS- and MDR-TB and BPaL for pre-XDR- and XDR-TB.*

*As some questions are only relevant for certain stakeholders, there will be different versions compiled of the semi-structured interview and FGD guide**. Please inform the interviewee(s) on the novel regimens BPaMZ and BPaL on trial by the TB Alliance and the purpose of this research.*

| *Please read out the following section:*  Good day, my name is ____________ and I am a PMDT consultant. I will interview you on behalf of the TB Alliance on the current TB treatment and novel regimens BPaMZ and BPaL, on trial by the TB Alliance. Before we start with the interview I will give a brief presentation to provide you with information on the novel regimens. More information on these and the current regimens can be found in the background information document. |
| --- |

*Give the presentation on BPaMZ and BPaL.*

| *Please read out the following section:*  During this interview/focus group discussion I will ask you questions on your perceptions on current TB management and how you would anticipate the possible implementation of the novel regimens into your country. We will discuss the benefits and challenges of the current regimens for DS- and DR-TB in comparison to the novel regimens BPaMZ and BPaL, on trial by the TB Alliance. At the end I will ask you to complete an acceptability matrix and a likelihood of implementation score. These will assess how acceptable you perceive the aspects of clinical and programmatic management of the current- and novel-TB regimens and how likely you would implement the novel regimens.  Your answers will be audio-recorded and notes will be taken.  You can refuse to answer any question or to stop the interview at any time. All the information you provide will be anonymized for publication. All data collected need to be stored in locked cabinets in the KNCV office. Access will be limited to TBA project staff.  Do you have any questions about the study?  Please read the informed consent form and sign if you agree to participate in this study. |
| --- |

# **Semi-structured interview/FGD:**

**Instructions**  *Please make sure to collect the signed informed consent(s)* ***before*** *proceeding with the semi-structured interview/FGD. Only proceed if signed consent has been given.*

*Please follow the instructions as indicated below throughout the semi-structured interviews/ FGDs:*

1. *Introduce each aspect of TB management by referring to the background information document.*
2. *Review with the interviewees whether the provided information in the background information document on current TB management is correct.*
3. *Continue with the questions on the respective aspect.*
4. *Please make notes of answers/keywords which you deem important.*

*Please collect at the end of the semi-structured interview/FGD the background information document on planning needs and acceptability of novel regimens in trial by the TB Alliance.*

*Please read out the following section:*

During this interview we will first discuss the clinical and programmatic management of DS-TB and DR-TB patients in your country. Thereafter, I would like to discuss the perceived benefits and challenges of the novel regimens BPaMZ and BPaL in comparison to the current TB management of DS- and DR- TB patients.

The following will be explored:

1. Treatment regimens, target groups and use in sub-populations
2. Diagnostic algorithm
3. Baseline assessment and monitoring of treatment efficacy
4. Monitoring of treatment safety
5. Patient friendliness
6. Programmatic aspects
7. Patient Support
8. Human Resources
9. Procurement and Supply Chain Management (PSCM)
10. Regimen Costs

*Please show* *Table 1. Overview of Clinical Trials on BPaMZ and BPaL*

## **Treatment regimens**

| *Please show: Background Information - Table 2. Overview of current and novel treatment regimens; Table 3. Target groups for current and novel treatment regimens and Table 4. Use of current and novel regimens in patient sub-populations*  *Part:* | | | | | | | |
| --- | --- | --- | --- | --- | --- | --- | --- |
| HRZE | RZELfx | Novel Regimen BPaMZ (4 months) | Shorter DR-TB Treatment Regimen (STR) | Novel Regimen BPaMZ (6 months) | Individualized Treatment Regimen (ITR) | Novel Regimen BPaL (Nix) | Novel Regimen BPaL (ZeNix) |
| **Questions**: How do you envision the distribution of novel and current regimens for different patient groups? | | | | | | | |

# **Diagnostic algorithm**

| **Current Diagnostic Algorithm** | **Novel Diagnostic Algorithm** |
| --- | --- |
| *Please show: Background Information -* *Table 5. Overview of current and proposed novel* *diagnostic/treatment algorithm part A: Current diagnostic/treatment algorithm* | *Please show: Background Information - Table 5. Overview of current and proposed novel diagnostic/treatment algorithm part A: Novel diagnostic/treatment algorithm* |
| **Questions:**  Is the novel diagnostic/treatment algorithm feasible/acceptable in your country? Explain why/why not.  If not, given levels of resistance to the drugs and diagnostic capacity in your setting, what algorithm would you propose for BPaMZ? | |

## **Baseline assessment and monitoring of treatment efficacy**

| *Please show: Background in*formation - Table 6. Baseline assessment and monitoring of treatment efficacy for the current and novel regimens  Part: | | | | | | | |
| --- | --- | --- | --- | --- | --- | --- | --- |
| HRZE | RZELfx | Novel Regimen BPaMZ (4 months) | Shorter DR-TB Treatment Regimen (STR) | Novel Regimen BPaMZ (6 months) | Individualized Treatment Regimen (ITR) | Novel Regimen BPaL (Nix) | Novel Regimen BPaL (ZeNix) |
| **Questions**:   1. In practice what types of treatment response monitoring are provided for patients with DS-TB, Isoniazid-mono resistant TB and Rifampicin-resistant TB without additional resistance to Fluoroquinolones? 2. Which benefits and challenges do you anticipate for BPaMZ in comparison to the current regimens in terms of treatment response monitoring?   Think of e.g.:   - Types and frequency of tests required - Accessibility of tests (e.g. level of care, out-of-pocket costs, patient travel)  1. What changes would be needed in terms of response monitoring if you were to have BPaMZ as a single treatment for DS-TB, Isoniazid-mono resistant TB and Rifampicin-resistant TB without additional resistance to Fluoroquinolones? | | | | | 1. In practice what types of treatment response monitoring are provided for patients with patients with Fluoroquinolone-resistant TB? 2. Which benefits and challenges do you anticipate for BPaL in comparison to the current regimens in terms of treatment response monitoring?   Think of e.g.:   - Types and frequency of tests required - Accessibility of tests required (e.g. level of care, out-of-pocket costs, patient travel) | | |

## **Monitoring of treatment safety**

| *Please show: Background In*formation - Table 7. Monitoring of treatment safety of the current and novel regimens  Part: | | | | | | | |
| --- | --- | --- | --- | --- | --- | --- | --- |
| HRZE | RZELfx | Novel Regimen BPaMZ (4 months) | Shorter DR-TB Treatment Regimen (STR) | Novel Regimen BPaMZ (6 months) | Individualized Treatment Regimen (ITR) | Novel Regimen BPaL (Nix) | Novel Regimen BPaL (ZeNix) |
| **Questions**:   1. In practice what types of treatment safety monitoring are provided for patients with DS-TB, Isoniazid-mono resistant TB and Rifampicin-resistant TB without additional resistance to Fluoroquinolones? 2. Which benefits and challenges do you anticipate for BPaMZ in comparison to the current regimens in terms of treatment safety monitoring?   Think of e.g.:   - Types and frequency of tests required - Accessibility of tests (e.g. level of care, out-of-pocket costs, patient travel) - Ancillary drugs  1. What changes would be needed in terms of safety monitoring if you were to have BPaMZ as a single treatment for DS-TB, Isoniazid-mono resistant TB and Rifampicin-resistant TB without additional resistance to Fluoroquinolones? | | | | | 1. In practice what types of treatment safety monitoring are provided for patients with Fluoroquinolone-resistant TB? 2. Which benefits and challenges do you anticipate for BPaL in comparison to the current regimens in terms of treatment safety monitoring?   Think of e.g.:   - Types and frequency of tests required - Accessibility of tests (e.g. level of care, out-of-pocket costs, patient travel) - Ancillary drugs | | |

## **Patient friendliness**

| *Please show: Background In*formation - *Table 8. Overview of patient friendliness and programmatic aspects of use of current and novel regimens*  Part: | | | | | | | |
| --- | --- | --- | --- | --- | --- | --- | --- |
| HRZE | RZELfx | Novel Regimen BPaMZ (4 months) | Shorter DR-TB Treatment Regimen (STR) | Novel Regimen BPaMZ (6 months) | Individualized Treatment Regimen (ITR) | Novel Regimen BPaL (Nix) | Novel Regimen BPaL (ZeNix) |
| **Questions**:   1. Which benefits and challenges do you anticipate for BPaMZ in comparison to the current regimen in terms of patient friendliness for patients with DS-TB, Isoniazid-mono resistant TB and Rifampicin-resistant TB without additional resistance to Fluoroquinolones?   Think of e.g.:   - Treatment duration - Side effects - Ease of administration (Pill burden, formulation, dosing no use of injectables - Patient out of pocket costs - Accessibility of care (e.g. distance, travel cost, time) - Treatment adherence  1. What implications would there be on patient friendliness if you were to have BPaMZ as a single treatment for DS-TB, Isoniazid-mono resistant TB and Rifampicin-resistant TB without additional resistance to Fluoroquinolones? | | | | | 1. Which benefits and challenges do you anticipate for BPaL in comparison to the current regimens in terms of patient friendliness for patients with Fluoroquinolone-resistant TB?   Think of e.g.:   - Treatment duration - Side effects - Ease of administration (Pill burden, formulation, dosing no use of injectables - Patient out of pocket costs - Accessibility of care (e.g. distance, travel cost, time) - Treatment adherence | | |

## **Programmatic aspects**

| *Please show: Background In*formation - *Table 8. Overview of patient friendliness and programmatic aspects of use of current and novel regimens*  Part: | | | | | | | |
| --- | --- | --- | --- | --- | --- | --- | --- |
| HRZE | RZELfx | Novel Regimen BPaMZ (4 months) | Shorter DR-TB Treatment Regimen (STR) | Novel Regimen BPaMZ (6 months) | Individualized Treatment Regimen (ITR) | Novel Regimen BPaL (Nix) | Novel Regimen BPaL (ZeNix) |
| **Questions**:   1. Which benefits and challenges do you anticipate for BPaMZ in comparison to the current regimen in terms of programmatic aspects for patients with DS-TB, Isoniazid-mono resistant TB and Rifampicin-resistant TB without additional resistance to Fluoroquinolones?   Think of e.g.:   - Regimen design - Level of care for treatment initiation - Hospitalization requirements - DOT requirements - Treatment duration - Frequency of administration - Treatment administration - FDC compatibility  1. What changes would be needed in terms of treatment modality if you were to have BPaMZ as a single treatment for DS-TB, Isoniazid-mono resistant TB and Rifampicin-resistant TB without additional resistance to Fluoroquinolones? | | | | | 1. Which benefits and challenges do you anticipate for BPaL in comparison to the current regimens in terms of treatment modality for patients with Fluoroquinolone-resistant TB? | | |

## **Patient Support**

| **Questions**:   1. What kind of patient support is provided for patients with DS-TB, Isoniazid-mono resistant TB and Rifampicin-resistant TB without additional resistance to Fluoroquinolones? 2. Which benefits and challenges do you anticipate for BPaMZ in comparison to the current regimen in terms of patient support? 3. What changes would be needed in terms of patient support if you were to have BPaMZ as a single treatment for DS-TB, Isoniazid-mono resistant TB and Rifampicin-resistant TB without additional resistance to Fluoroquinolones?   Think of e.g.:   - Eligibility criteria - Type of support provided (e.g. food, transportation, other financial subsidies etc.) - Duration of support - Potential partners (GF etc.) | 1. What kind of patient support is provided for patients with Fluoroquinolone-resistant TB? 2. Which benefits and challenges do you anticipate for BPaL in comparison to the current regimens in terms of patient support? |
| --- | --- |

## **Human Resources**

| **Questions:**   1. Which benefits and challenges do you anticipate for BPaMZ in comparison to the current regimens in terms of human resource management for patients with DS-TB, Isoniazid-mono resistant TB and Rifampicin-resistant TB without additional resistance to Fluoroquinolones?   Think of e.g.:   - Staff capacity/ workload - Staff requirements (number of staff and training needs) - Supervision  1. What changes would be needed in terms of human resources if you were to have BPaMZ as a single treatment for patients with DS-TB, Isoniazid-mono resistant TB and Rifampicin-resistant TB without additional resistance to Fluoroquinolones?   Think of e.g.:   - Task-shifting to lower levels of the health system - Work load and capacity requirements at lower levels of system | 1. Which benefits and challenges do you anticipate for BPaL in comparison to the current regimens in terms of human resource management for patients with Fluoroquinolone-resistant TB?   Think of e.g.:   - Staff capacity/ workload - Staff requirements (number of staff and training needs) - Supervision |
| --- | --- |

## **Procurement and Supply Chain Management (PSCM)**

| *Please show: Background in*formation sheet – *Table 9. Overview of procurement and supply chain management, drug costs and child friendly formulations for the current and novel regimen*  Part: | | | | | | | |
| --- | --- | --- | --- | --- | --- | --- | --- |
| HRZE | RZELfx | Novel Regimen BPaMZ (4 months) | Shorter DR-TB Treatment Regimen (STR) | Novel Regimen BPaMZ (6 months) | Individualized Treatment Regimen (ITR) | Novel Regimen BPaL (Nix) | Novel Regimen BPaL (ZeNix) |
| **Questions:**   1. Which benefits and challenges do you anticipate for BPaMZ in comparison to the current regimens for in terms of PSCM for patients with DS-TB, Isoniazid-mono resistant TB and Rifampicin-resistant TB without additional resistance to Fluoroquinolones?   Think of e.g.:   - Quantification and forecasting process (data availability, calculations, avoiding over and under stocks) - Wastages, shortages, emergency orders  1. What changes would be needed in terms of PSCM if you were to have BPaMZ as a single treatment for patients with DS-TB, Isoniazid-mono resistant TB and Rifampicin-resistant TB without additional resistance to Fluoroquinolones?   Think of e.g.:   - Procurement, forecasting and supply chain processes - Administrative costs of arranging procurement for multiple formulations and regimens - Funding sources and channels for DS- vs. DR-TB | | | | | 1. Which benefits and challenges do you anticipate for BPaL in comparison to the current regimens in terms of PSCM for patients with Fluoroquinolone-resistant TB?   Think of e.g.:   - Quantification and forecasting process (data availability, calculations, avoiding over and under stocks) - Wastages, shortages, emergency orders | | |

# **Regimen Costs**

| *Please show: Background in*formation sheet – *Table 9. Overview of procurement and supply chain management, drug costs and child friendly formulations for the current and novel regimen*  Part: | | | | |
| --- | --- | --- | --- | --- |
| HRZE | RZELfx | Novel Regimen BPaMZ (4 months) | Shorter DR-TB Treatment Regimen (STR) | Novel Regimen BPaMZ (6 months) |
| **Question:**   1. What would be an acceptable threshold price for BPaMZ as a single treatment for patients with DS-TB, Isoniazid-mono resistant TB and Rifampicin-resistant TB without additional resistance to Fluoroquinolones? | | | | |

**note: a costing study will still be done to assess the potential costs/cost savings of the regimen.*

*Please collect at the end of the semi-structured interview/FGD the background information document on planning needs and acceptability of novel regimens in trial by the TB Alliance.*
