# Supplementary material for: Acceptability, feasibility, and likelihood of stakeholders implementing the novel BPaL regimen to treat extensively drug-resistant tuberculosis patients
Source: BMC Public Health. 2021 Jul 16;21:1404. doi: 10.1186/s12889-021-11427-y (PMC8284025; doi:10.1186/s12889-021-11427-y)
Supplement: Supplementary file 3 — Additional file 3. [file 12889_2021_11427_MOESM3_ESM.docx]

# **Acceptability matrix and overall likelihood of implementing the novel regimens BPaMZ and BPaL**

Please indicate in the matrix below how acceptable do you perceive the in the interview covered aspects of clinical and programmatic management of current and novel TB regimens on a scale from 1 (Very Unacceptable ) to 5 (Very Acceptable). Please give one score per cell.

| Very Unacceptable | Unacceptable | Neutral | Acceptable | Very Acceptable |
| --- | --- | --- | --- | --- |

| **1** | **2** | **3** | **4** | **5** |
| --- | --- | --- | --- | --- |

|  | **Current TB regimens** | | | | **Novel TB regimens** | | | | | |
| --- | --- | --- | --- | --- | --- | --- | --- | --- | --- | --- |
|  | **HRZE** | **HRZELfx** | **Shorter DR-TB Treatment Regimen (STR)** | **Individualized Treatment Regimen (ITR)** | **BPaMZ for DS- TB** | **BPaMZ as a single regimen for Isoniazid-mono resistant TB** | **BPaMZ for Rifampicin-resistant TB without additional resistance to Fluoroquinolones** | **BPaMZ as a single regimen for DS-TB, Isoniazid-mono resistant TB and Rifampicin-resistant TB without additional resistance to Fluoroquinolones** | **BPaL (Nix)** | **BPaL (ZeNix)** |
| **How acceptable do you perceive the current and the novel diagnostic algorithm?** |  | | | |  | | | | | |
| **How acceptable do you perceive the baseline assessment and monitoring of treatment efficacy requirements for each regimen?** |  |  |  |  |  |  |  |  |  |  |
| **How acceptable do you perceive the safety monitoring requirements for each regimen?** |  |  |  |  |  |  |  |  |  |  |
| **How patient friendly do you perceive each regimen?** |  |  |  |  |  |  |  |  |  |  |
| **How acceptable do you perceive each regimen in terms of programmatic aspects?** |  |  |  |  |  |  |  |  |  |  |
| **How acceptable do you perceive the patient support requirements for each regimen?** |  |  |  |  |  |  |  |  |  |  |
| **How acceptable do you perceive the human resource needs for each regimen?** |  |  |  |  |  |  |  |  |  |  |
| **How acceptable do you perceive the procurement and supply chain management requirements for each regimen?** |  |  |  |  |  |  |  |  |  |  |

Regarding the introduction and implementation of the novel regimens, overall, given what you understand about the relative benefits, challenges and trade-offs of different regimens, if approved by a stringent and/or national regulatory authority and WHO endorsed, how likely would you implement them?

| How likely would you implement BPaMZ as the Standard of Care (SoC) for treatment of DS-TB as a 4-month regimen?  ^1^⬜ Very unlikely ^2^⬜ Unlikely ^3^⬜ Neutral ^4^⬜ Likely ^5^⬜ Very Likely |
| --- |
| How likely would you implement BPaMZ as the SoC for treatment of DS-TB as a 3-month regimen?  ^1^⬜ Very unlikely ^2^⬜ Unlikely ^3^⬜ Neutral ^4^⬜ Likely ^5^⬜ Very Likely |
| *If you ticked “neutral”, “unlikely” or “very unlikely”:*  How likely would you implement BPaMZ in certain sub-populations (e.g. INH-r, TB/HIV, etc.). for treatment of DS-TB?  ^1^⬜ Very unlikely ^2^⬜ Unlikely ^3^⬜ Neutral ^4^⬜ Likely ^5^⬜ Very Likely  *If you ticked “likely” or “very likely”:* Which sub-populations? _______________________ |
| How likely would you implement BPaMZ as the SoC for treatment of Rifampicin-resistant TB without additional resistance to Fluoroquinolones?  ^1^⬜ Very unlikely ^2^⬜ Unlikely ^3^⬜ Neutral ^4^⬜ Likely ^5^⬜ Very Likely |
| *If you ticked “neutral”, “unlikely” or “very unlikely”:*  How likely would you implement BPaMZ in certain sub-populations (e.g. TB-HIV) for treatment of Rifampicin-resistant TB without additional resistance to Fluoroquinolones?  ^1^⬜ Very unlikely ^2^⬜ Unlikely ^3^⬜ Neutral ^4^⬜ Likely ^5^⬜ Very Likely  *If you ticked “likely” or “very likely”:* Which sub-populations? _______________________ |
| If the INH-r regimen by the WHO was implemented in your country, how likely would you adapt BPaMZ as alternative regimen for DS-TB?  ^1^⬜ Very unlikely ^2^⬜ Unlikely ^3^⬜ Neutral ^4^⬜ Likely ^5^⬜ Very Likely |
| How likely would you implement BPaMZ as the SoC for combined treatment of DS-TB, Isoniazid-mono resistant TB and Rifampicin-resistant TB without additional resistance to Fluoroquinolones?  ^1^⬜ Very unlikely ^2^⬜ Unlikely ^3^⬜ Neutral ^4^⬜ Likely ^5^⬜ Very Likely |
| How likely would you implement BPaL as the SoC for treatment of XDR-TB and MDR-TB treatment failure/intolerance based on initial profile (Nix)?  ^1^⬜ Very unlikely ^2^⬜ Unlikely ^3^⬜ Neutral ^4^⬜ Likely ^5^⬜ Very Likely |
| How likely would you implement BPaL as the SoC for treatment of Fluoroquinolone-resistant TB without additional resistance to second-line injectables based on initial profile (Nix)?  ^1^⬜ Very unlikely ^2^⬜ Unlikely ^3^⬜ Neutral ^4^⬜ Likely ^5^⬜ Very Likely |
| How likely would you implement BPaL as the SoC for treatment of XDR-TB and MDR-TB treatment failure/intolerance once dosing is simplified/optimized through ZeNix trial?  ^1^⬜ Very unlikely ^2^⬜ Unlikely ^3^⬜ Neutral ^4^⬜ Likely ^5^⬜ Very Likely |
| How likely would you implement BPaL as the SoC for treatment for Fluoroquinolone-resistant TB without additional resistance to second-line injectables once dosing is simplified and optimized (ZeNix)?  ^1^⬜ Very unlikely ^2^⬜ Unlikely ^3^⬜ Neutral ^4^⬜ Likely ^5^⬜ Very Likely |
